# The 2018 Astana Declaration on Primary Health Care, is it useful?

**DOI:** 10.7189/jogh.09.010313

**Published:** 2019-06

**Authors:** Gijs Walraven

**Affiliations:** Aga Khan Development Network, Geneva, Switzerland

40 years ago, health experts and policy makers from 134 countries convened in what is now Almaty to attend a conference on international primary health care. On 12 September 1978 the Alma-Ata Declaration was approved, with the ambitious goal of achieving “health for all by the year 2000” [1]. ‘Alma-Ata’s’ target of “health for all” was based on the World Health Organization’s constitutional definition, where health is “a state of physical, mental and spiritual well-being, not merely an absence of disease or infirmity” – a highly aspirational rather than measurable objective. According to Halfdan Mahler, the inspirational director-general of the World Health Organization at the time, Alma Ata was “one of the rare occasions where a sublime consensus between the haves and the have-nots in local and global health emerged” [2]. Its Declaration revolutionized the world’s interpretation of health with the core principles of universal access to care, equity, community participation, intersectoral collaboration, and appropriate use of resources, as well as by stating that inadequate and unequal health care was unacceptable: economically, socially, and politically [3]. These principles remain relevant today, but regrettably in many countries commitments have gone unfulfilled. This viewpoint argues that the outcomes of the 2018 Astana Declaration as a successor to Alma-Ata will also be deficient and health inequalities will persist unless specific actions are taken to truly put primary health care at the centre of the overall health system [4].

## THE LEGACY OF ALMA-ATA

With an emphasis on local ownership, primary health care honoured the resilience and ingenuity of the human spirit and made space for solutions created, owned and sustained by communities. Primary health care also offered a way to organize the full spectrum of health care, from households to hospitals, with prevention as important as cure, and with resources invested rationally in the different levels of care.

Unfortunately, the comprehensive approach proposed under Alma-Ata was almost immediately misunderstood; it was regarded by some as an attack on the medical establishment, and it was confused with an exclusive focus on first-level health care. For some it was regarded as cheap: poor care for poor people, a second rate solution for poor countries. And within a year of the Declaration, a simpler approach was proposed involving a more top down course of action and focussing on fewer interventions that were most justified by epidemiological importance and technical affordability. This more selective approach was considered to be more feasible, measurable, rapid, and less risky than really empowering communities to make choices. Interventions in the areas of maternal and child health, immunization, family planning and micro-nutrients were delivered through “vertical” programmes, which took the decisions out of the hands of communities, but rapidly reached high coverage for the selected priorities. One can argue that the health-related Millennium Development Goals or MDGs were developed in line with this selective approach.

The goal of “health for all” by the year 2000 was not met. When asked about the meaning of the “health for all” goal, Halfdan Mahler said “the goal was not to eradicate all diseases and illnesses by 2000; we knew that would have been impossible. Our goal was to focus world attention on health inequalities and on trying to attain an acceptable level of health, equitably distributed throughout the world” [2]. Although the goal of “health for all” remains unfulfilled in much of the world, there are countries with primary health care inspired by Alma-Ata with strong evidence of better population health outcomes and reduced inequalities at lower cost. Examples include Chili, Cuba, Ethiopia, Nepal, Rwanda and Sri Lanka [3,5,6].

## THE ASTANA DECLARATION

In October 2018, 2000 delegates from more than 120 countries renewed the commitment to comprehensive primary health care for all with the Astana declaration. The new primary health care declaration affirms the “commitment to the fundamental right of every human being to the enjoyment of the highest attainable standard of health without distinction of any kind”, and reaffirms the commitment to the Alma-Ata core principles [4]. The Astana Declaration recognizes that remaining healthy is challenging for many people, particularly the poor, and states that it is “unacceptable that inequity in health and disparities in health outcomes persist”.

‘Astana’ commits itself to prioritizing disease prevention and health promotion and aims to meet all people’s health needs across the life course through comprehensive preventive, promotive, curative, rehabilitative services and palliative care. The new Declaration recognizes the increasing importance of non-communicable diseases including mental health issues, injuries and the health impacts of climate change. Further, Astana incorporates ‘universal health coverage’ that is at the centre of the health related Sustainable Development Goal (SDG) 3 [7]. The 2015 SDGs that succeeded the MDGs can provide impetus to the Alma Ata and Astana principles through other SDGs such as ‘equity’(SDG 10), ‘community participation’ (SDG 6) and ‘intersectoral collaboration’ (SDG 17), while evidence shows that countries that reorient their health systems towards primary care are better placed to achieve almost all of the SDGs, including SDG 1 – ‘end poverty’[8,9].

## LACK OF SPECIFICS

Although the Astana Declaration reconfirms the solidarity of commitment to the right of health for all enshrined in the Alma-Ata Declaration and the desire to achieve it, the specific actions needed to make this vision a reality are as absent as they were in 1978. In a well-functioning health system there are health promotion and prevention activities at the community level, and a patient will go in the first instance to a primary health care centre. Only if that clinic lacks the skills and equipment to treat the patient will they go to a next level of health service delivery, and the same applies for further levels. The system is advantageous for the patient and relatives, as primary health care facilities can be small and therefore within reasonable travel distance. This system is also economical because it should lead to expensive skills and equipment being used only on patients who need it. If a patient who should have been treated at a first level referral community or district hospital is admitted to a next level referral care hospital, more expensive diagnostic tests are likely to be used to confirm the diagnosis of a common condition, simply because the facilities are available.

In practice, in many countries this system does not work as intended. A high proportion of patients treated in a hospital could have been treated in a primary health care centre. This is partly because the patient knows that the hospital has better facilities and has doctors with specialized skills. The patient thus goes direct to its outpatient department in the hope of the 'best' treatment, bypassing primary care. It may also happen because it is known that the hospital hardly ever runs out of supplies, particularly of drugs, while this is too often the case at lower levels of the health care system. Thus hospitals become used by local, often more affluent urban patients with common conditions. The costs of treating these common conditions are much higher at the hospital than they would have been at a primary health care clinic.

The availability of greatly expanded specialist diagnostic and therapeutic options due to the growth of medical technologies, and health financing policies that free patients of financial constraints to seeking hospital-based specialized services is resulting in unnecessary complex care in preference to preventive services or routine consultations. Many of the ills of health care systems reflect an overreliance on advanced medical technology and an overestimation of the benefits of cure, rather than prevention of disease or the promotion of health. Worrisome trends include health systems that focus disproportionally on a narrow band of specialized curative care, and where a hands-off approach to governance has allowed unregulated commercialization of health care to flourish.

If Astana is to be realised, lower-income countries should not make the same mistake as the one made by many high-income countries. The statement in the Astana Declaration to “strive to avoid fragmentation and ensure a functional referral system between primary and other levels of care” is in this regard important [6]. To move this forward requires strong political commitment and leadership that places primary health care at the centre of efforts to attain universal health coverage and governance structures and policy frameworks in support of primary health care. It also requires a participatory approach that empowers people and communities to play an active role in shaping the policies and a whole-of-government approach beyond Ministries of Health [10].

Specific actions to make Astana successful should promote major shifts in the relationship between health care providers and populations. This is a major difference between a narrow view of first-level ‘contact’ health services and the broader perspective of primary health care. Unfortunately, since 1978, in many countries the balance has tilted towards personal health care at the expense of population health. If this is not addressed the odds of Astana to succeed are low.

Health care of the person should take into account the influences and context of the community [11]. An orientation to the population requires the facility based primary health care worker to understand the local health problems and their social determinants, plan the most effective preventative and therapeutic interventions for the community through co-creation, and advocate for improved living conditions and ‘quality of life’, while still providing individual health care [12]. Community health workers can help, and play a vital role in such a primary health care system, especially if they work in tandem with facility-based health staff [13].

**Figure:**
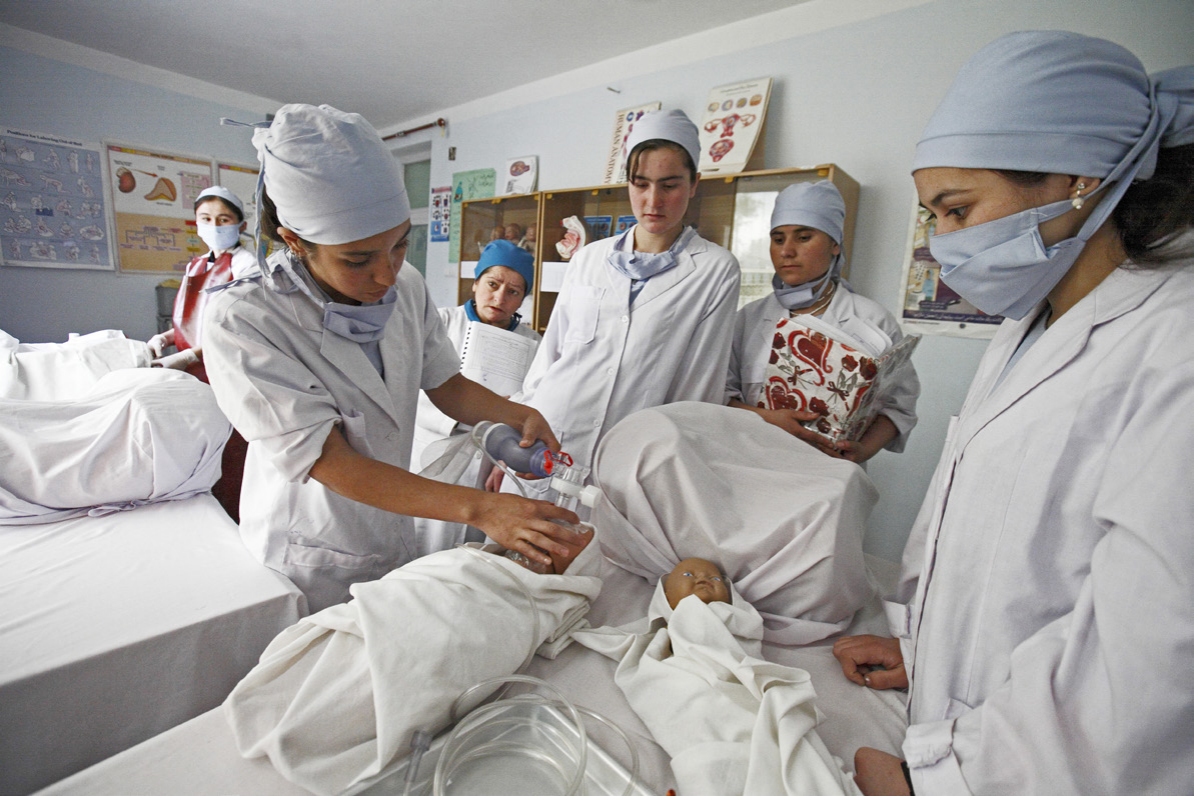
Photo: Community midwifery school in Badakhshan province, Afghanistan (Aga Khan Development Network/ Sandra Calligaro).

The vision is to assure that high-quality services are provided on the basis of a defined population, through proactive strategies, favouring continuity of care and focused attention for disease prevention and health promotion, guaranteeing an explicit and affordable set of evidence-based entitlements [14]. The introduction of a Basic Package of Health Services in Afghanistan is a recent example of translating such a vision into a practical set of actions [15]. Better measurement and sharing of effective models and practices has good potential to accelerate improvements in primary health care quality and effectiveness, and the Primary Health Care Performance Initiative (PHCPI) can play an important role in this [16]. In such actionable initiatives may lie the key to finally unlocking the full potential of Alma-Ata, and reach “health for all”, including, and especially, the poor.
